# Dr. William H. Gobrecht

**Published:** 1901-08

**Authors:** 


Dr. William H. Gobrecht, of Washington, D.C., died in that city July 19, 1901, aged 72 years. He was surgeon of the 49th Pennsylvania Volunteer Infantry during the civil war, and served for a portion of the time as surgeon-in-chief of Hancock's brigade in the second division (Smith's) of the sixth army corps. Dr. Gobrecht was an accomplished surgeon and an anatomist and edited the first editions of Wilson's Anatomy.

